# The Positive Influence of Polyphenols Extracted From *Pueraria lobata* Root on the Gut Microbiota and Its Antioxidant Capability

**DOI:** 10.3389/fnut.2022.868188

**Published:** 2022-03-29

**Authors:** Xiao Xu, Ying Guo, Shaoqin Chen, Wenliang Ma, Xinlei Xu, Shuning Hu, Lifang Jin, Jianqiu Sun, Jian Mao, Chi Shen

**Affiliations:** ^1^School of Life Sciences, Shaoxing University, Shaoxing, China; ^2^National Engineering Research Center for Cereal Fermentation and Food Biomanufacturing, Jiangnan University, Wuxi, China

**Keywords:** *Pueraria lobata*, antioxidant, polyphenols, gut microbiota, mice model

## Abstract

*Pueraria lobata*, an edible food and medicinal plant, is a rich source of bioactive components. In this study, a polyphenol-rich extract was isolated from *P. lobata*. Puerarin was identified, and the high antioxidant bioactivity of the *P. lobata* extract was evaluated using the methods of 2,2-diphenyl-1-picrylhydrazyl (DPPH), 2,2′-azinobis-(3-ethylbenzthiazoline-6-sulphonate) (ABTS), and hydroxyl free radical scavenging ratio. Additionally, the IC_50_ values of DPPH, ABTS, and hydroxyl radical scavenging activities were 50.8, 13.9, and 100.4 μg/ml, respectively. Then, the *P. lobata* extract was administered to C57Bl/6J mice and confirmed to have a superior effect on enhancing the antioxidant status including improving superoxide dismutase activity, glutathione peroxidase peroxide activity, total antioxidant capacity activity, and malondialdehyde contents *in vivo*. Furthermore, the *P. lobata* extract had beneficial and prebiotic effects on the composition and structure of gut microbiota. Results showed that the *P. lobata* extract significantly increased the abundance of beneficial bacteria, involving *Lactobacillaceae* and *Bacteroidetes*, and decreased the abundance of *Ruminococcaceae*, *Prevotellaceae*, and *Burkholderiaceae*. Overall, our results provided a basis for using the *P. lobata* extract as a promising and potential functional ingredient for the food industry.

## Introduction

*Pueraria* is a typical representative genus comprising over 20 species of herbs, which are highly found in China, Japan, and Korea. Among them, kudzu [*Pueraria montana* var. *lobata* (Willd.)] was first documented in a Chinese Materia Medica named Shen Nong Ben Cao Jing written in the Western Han Dynasty, and its medical properties have a long history of wide application in traditional Chinese medicine, as well as being developed as a royal special food for edible uses in Japanese cuisine (1, 2). *Pueraria lobata* has also been utilized to manage cardiovascular diseases, alcohol-induced liver injury, and many chronic diseases. Likewise, being an herbal, foodstuff could be widely used in nutritious foods and dietary supplements. According to preliminary statistics, the total area of cultivated and wild *P. lobata* in China is 400,000 hectares, with an annual production of more than 1.5 million tons. In the future decade, the global demand for kudzu is estimated to increase sharply to 50 million tons per year.

Polyphenols derived from food and medicinal plants have been known to prevent many chronic diseases, such as diabetes, atherosclerosis, and obesity (3). The major constituents of kudzu (the root of *P. lobata*) were high levels of starch, but recent studies showed that the extracts of *P. lobata* contained rich polyphenols, such as puerarin, genistin, daidzin, and daidzein. The high levels of polyphenolic compounds in *P. lobata* have recently attracted much attention due to the functional properties of lowering cholesterol, inhibiting cancer cell proliferation and tumor growth, and modulating the immune system (4). Some studies focus on the protective effect of puerarin on alcoholic liver disease, for instance, reducing the production of free radicals, improving the activities of antioxidant enzymes, stimulating AMPK and Nrf2 signaling pathways, inhibiting the mTOR, regulating the activities of ethanol dehydrogenase and aldehyde dehydrogenase, as a result of keeping the balance of cellular redox state, and promoting liver energy metabolism dysfunction. In the process of oxidative stress on the diet-induced obesity model, daidzein in *P. lobata* is associated with reduction of apoptosis and stearoyl-CoA desaturase 1, which is a key enzyme relative to obesity (5). Furthermore, *P. lobata* has been demonstrated to possess the capability of regulating inflammatory factors and pathways, resulting in exhibiting potential anti-inflammatory activity. Some studies also found that the biological properties of antioxidant and antimicrobial activities displayed by the *P. lobata* extract were attributed to the existence of phenolic compounds (6, 7).

The gut microbiota is a microbial community in the intestinal with over 100 trillion microorganisms, which is the largest and most complex ecosystem in the human body, and has been shown the participation in the body’s nutrient metabolism, immune system, and overall health of the host (8). A healthy gut, as a result of stable gut microbiota and diverse ecosystem, played an active influence in regulating metabolic pathways and resisting pathogen invasion (9). In contrast, if the gut ecosystem was broken and suffered from an imbalance of gut microbiota, a great deal of host health problems would be induced ceaselessly, including obesity, metabolic syndrome, diabetes, and intestinal bowel disease (10–12). In recent studies, the gut microbiota has been widely considered as a key role in the modulation of transformation of nutrients and compounds in the dietary supplements. Likewise, bioactive components in functional foods give an effect on gut microbiota. In fact, a novel potential therapy, based on altering the gut microbiota, aroused worldwide concern, especially in the treatment of preventing metabolic disorders (13). The abundance and diversity of gut microbiota could be examined using next-generation sequencing analysis of 16S rRNA amplicons to get knowledge about the profile of microbial characterization influenced by bioactive components. Liu et al. found that the promotion of *Bacteroides vulgatus*, *Parabacteroides distasonis*, *Prevotella*, and *Bifidobacterium* is a master regulator of anti-obesity properties (14). Additionally, the abundance of the *Prevotella* and *Akkermansia* genus could be increased by the flavan-3-ols, which was reported to be reversely linked to body weight gain and adipose tissue inflammation. Therefore, it is notable that the presence of specific gut bacterial species would participate in the improvement of the healthy gut ecosystem and cellular antioxidant state.

Pervious study suggested that *P. lobata* extract was closely related to antioxidant, antiproliferative, and anti-inflammation effects *via* cell models (15). However, few studies paid attention to elucidate the relationship and mechanism of the effect of *P. lobata* extract on the antioxidant activities and the composition of gut microbiota. In addition, there is a need to characterize a functional *P. lobata* extract and demonstrate its bioactive properties *in vitro* and *in vivo*. Therefore, in this study, we had a polyphenol-rich *P. lobata* extract, investigated the antioxidant *in vitro* and *in vivo*, as well as profiled the specific gut microbiota modulated by the polyphenol-rich extract. The health benefits of *P. lobata* extract could be better understood according to the direct comparison of microbial composition and structure *via* treated by *P. lobata* extract.

## Materials and Methods

### Materials and Chemicals

*Pueraria lobata* was purchased from a local market (Hangzhou, China). Then, the fruiting bodies of *P. lobata* root were grounded into powder and stored at a low-temperature refrigerator before being used. The antioxidant indicator kits of superoxide dismutase activities (SOD), glutathione peroxidase peroxide (GSH-Px) activities, total antioxidant capacity (T-AOC) activities, and malondialdehyde (MDA) contents were obtained from the Nanjing Jiancheng Bioengineering Institute (Nanjing, China). The other chemicals and solvents were of liquid chromatography grade and purchased from Fisher Scientific or Beijing Solarbio Science & Technology Limited Company (Beijing, China).

### Polyphenol-Rich Extract

The extraction method of *P. lobata* root was conducted according to a previous study with some modifications (16). In brief, *P. lobata* powder (10 g) was mixed with chilled acetone (200 ml) with ultra-sonicated for 30 min and then centrifuged for 20 min with the speed of 14,000 × *g* at 4°C. Then, the supernatant was removed, and the remaining pellet was re-extracted with another chilled acetone (200 ml). The pooled extracts were evaporated to dryness at 35°C. The macroporous resin column was used for the preliminary purification. The solution was then reconstituted in the solution of methanol, dried, and stored at −20°C before the test. The residue in this study referred to the *P. lobata* extract.

### Determination of Total Phenolic Content

The total phenolic content of the *P. lobata* extract was measured based on the method described previously with some minor modifications (17). The *P. lobata* extract was dissolved in methanol to a concentration of 0.25 mg/ml. Gallic acid standards or the samples (20 μL) and diluted Folin–Ciocalteu reagent were incubated at room temperature for 1 min, followed by terminating reaction with sodium carbonate (75 mg/ml). Molecular Devices SpectraMax microplate spectrophotometer (San Jose, CA, United States) was used to record the absorbance at 760 nm. Total phenolic contents were expressed as μmol gallic acid equivalents/mg polyphenol-rich *P. lobata* extract.

### High-Performance Liquid Chromatography Analysis

The chromatographic analysis of the *P. lobata* extract was performed using a high-performance liquid chromatography (HPLC) system (Waters, Milford, MA, United States) (18, 19). The mobile phases were prepared as follows: water with 0.1% formic acid and acetonitrile with 0.1% formic acid. The gradient procedure was as follows: 0–3 min, 5% B, 3–42 min, 5%–35% B, 42–46 min, 35%–90% B, 46–50 min, 90% B, 50–56 min, 90%–5% B. The column was maintained at 30°C. The flow rate was set at 0.8 ml/min. The *P. lobata* extract was separated using an Agilent ZORBAX Eclipse Plus C18 (250 mm × 4.6 mm, 5.0 μm particle size). The detection of the *P. lobata* extract was recorded using an ultraviolet detector at 280 nm wavelength.

### Antioxidant Activities

#### 2,2-Diphenyl-1-Picrylhydrazyl Free Radical Scavenging Activity

The 2,2-diphenyl-1-picrylhydrazyl (DPPH) assay solution was performed according to a previous study prepared by mixing samples or blank solution (20 μl) with 50% ethanol solution containing DPPH solution (180 μl) (20). The mixture was incubated at room temperature for 60 min in the dark. The absorbance was measured at 517 nm. The results were described using the DPPH scavenging ratio, which was calculated as follows:


$$\mathrm{The}\mathrm{DPPH}\mathrm{scavenging}\mathrm{ratio}\left(\%\right)=\left[1-A_{\text{sample}}/A_{\text{control}}\right]\times 100$$

*A*_control_ is the absorbance measured for blank, and *A*_sample_ is the absorbance measured for the *P. lobata* extract. Ascorbic acid (vitamin C) was selected as the positive control.

#### 2,2′-Azinobis-(3-Ethylbenzthiazoline-6-Sulphonate) Free Radical Scavenging Activity

The 2,2′-azinobis-(3-ethylbenzthiazoline-6-sulphonate) (ABTS) scavenging activity was measured based on the method studied described previously with minor modification (21). ABTS^⋅⁣ +^ was generated by mixing 7 mM of ABTS solution with a 2.45 mM of the aqueous solution of K_2_S_2_O_8_ and reacting for 12 h at room temperature in the dark. The ABTS^⋅⁣ +^ reaction solution (100 μl) with an absorbance of 0.70 cm^–1^ at 734 nm was used, and 100 μl of samples or blank solution was mixed and reacted for 60 min in the dark. The absorbance was recorded at 734 nm. The results were shown by the ABTS^⋅⁣ +^ scavenging ratio, which was calculated as follows:


ABTSscavenging⋅+ratio(%)=[1-Asample/Acontrol]×100

*A*_control_ is the absorbance of water and *A*_sample_ is the absorbance of samples. Ascorbic acid (vitamin C) was used as a positive control.

#### Hydroxyl Radical Scavenging Activity

The hydroxyl radical scavenging activity was measured based on the method reported in a previous study (21). In brief, the *P. lobata* extract solution (100 μl) was mixed with 100 μl of 6 mM ferrous sulfate solution and 6 mM salicylic acid ethanol solution. The mixtures were mixed with 1 ml of 0.1% hydrogen peroxide and reacted for 30 min in the dark. The absorbance was recorded at 510 nm, and the results were displayed by the hydroxyl radical scavenging ratio.

### Mice Animal Design

The animal experimental protocols were approved and authorized by the Institutional Animal Care and Use Committee of China Pharmaceutical University (SYXK-2020-0023). The experimental test was designed according to the study with minor modifications (22). Twelve specific pathogen-free male C57BL/6 mice (6 weeks of age, around 18 g) were purchased from the Model Animal Research Center of Nanjing University. The mice were divided randomly into two groups after acclimatization for 1 week. In brief, except for daily gavage with water, the two-group mice were separately fed by the normal diet with or without *P. lobata* extract (200 mg/kg/day). The body weight of each mouse was measured daily. Also, the feces were collected at the end of the feeding duration and the experimental period. The feces of two experimental groups were immediately frozen in liquid nitrogen and stored at −80°C before the sequence analysis. All the animals were sacrificed using isoflurane as an anesthetic. Livers from each mouse were excised and collected for biochemical assays.

### The Antioxidant Status of Mice Liver

The biochemical parameters of liver samples from the mice, including SOD, GSH-Px activity, T-AOC, and MDA activity, were prepared according to the study and determined by using commercial kits following the manufacturer’s instructions (23).

### The Sequencing of Gut Microbiota

Total genome DNA was extracted from the fecal samples of the control and the *P. lobata* extract groups using a QIAamp DNA stool kit, and the DNA concentration and purity were monitored on 1% agarose gels, and then diluted to 1 ng/μl for amplicon generation. The 16S V3-V4 rDNA genes were amplified using the specific primer with the barcode. All PCR reactions were carried out with the high-fidelity PCR master mix, forward and reverse primers, and template DNA. Thermal cycles were conducted as the following: initial denaturation at 98°C for 1 min, 30 cycles of denaturation at 98°C for 10 s, annealing at 50°C for 30 s, and elongation at 72°C for 5 min. Then, the PCR products were mixed and purified using the Axygen AxyPrep DNA Gel Extraction Kit. Finally, the sequencing libraries were generated using the NEBNext^®^ Ultra™ DNA Library Prep Kit following the manufacturer’s instructions and then sequenced on an Illumina MiSeq platform (San Diego, CA, United States).

### Gut Microbiota Data Analysis

The paired-end reads from the original DNA fragments from the control and the *P. lobata* extract groups were merged using the Flash software. The aired-end reads were assigned by the different barcodes. Then, the Upare software package was used to perform the sequences. The alpha and beta diversities were also analyzed by the In-house Perl scripts. The sequences with ≥97% similarity were assigned to the same operational taxonomic units (OTUs). The observed species were used to estimate the amount of unique OTUs, as well as the rarefaction curves were generated based on these three metrics. The cluster analysis was illustrated using the principal component analysis and the principal coordinate analysis (PCoA). The Stamp software was utilized to study the differences in the abundances of individual taxonomy. The Linear discriminant analysis Effect Size (LEfSe) was used for the quantitative analysis of biomarkers among different groups. The 16S rRNA raw sequence data generated were deposited into the National Center for Biotechnology Information Sequence Read Archive Sprout and available under the accession number of PRJNA805070^1^.

### Statistical Analysis

The SPSS software (version 24.0) and excel (version 2020) were used for statistical analysis. The Student’s *t*-test was used to assess the mean difference between the *P. lobata* extract and the control groups. A *p*-value of <0.05 or <0.01 was considered statistically significant. The results were expressed as the mean ± standard deviation.

## Results and Discussion

### Identification of Polyphenolic Compounds in the *P. lobata* Extract

**FIGURE 1:**
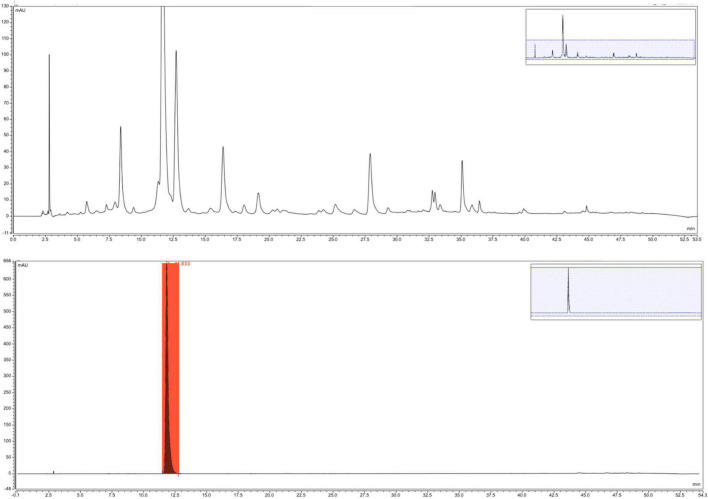
Phenolic high-performance liquid chromatography (HPLC) spectra of the *Pueraria lobata* extract and compound puerarin.

*Pueraria lobata* has been in the Chinese List of Food and Medicine Homologous Substance since 2002 and is currently regarded as a functional food in the food industry due to possessing a great number of bioactive compounds such as isoflavones, polysaccharides, terpenoids, and amino acids. It has been reported that the antioxidant activity of the *P. lobata* extract was well correlated with the content of phenolic compounds (24). Although other antioxidants were probably present in the *P. lobata* extract, phenolic compounds made a significant contribution to the antioxidant activity. The polyphenol-rich *P. lobata* extract (EPM) was eluted by gradient elution HPLC (Figure 1). The yield content of this *P. lobata* extract is 1.2%. The total phenolic content of this extract was 67.3 mg gallic acid equivalents/g dry weight.

### Effect of the *P. lobata* Extract on Antioxidant Activity Analysis

#### Effect of the *P. lobata* Extract on 2,2-Diphenyl-1-Picrylhydrazyl Radical Scavenging Activity

**FIGURE 2:**
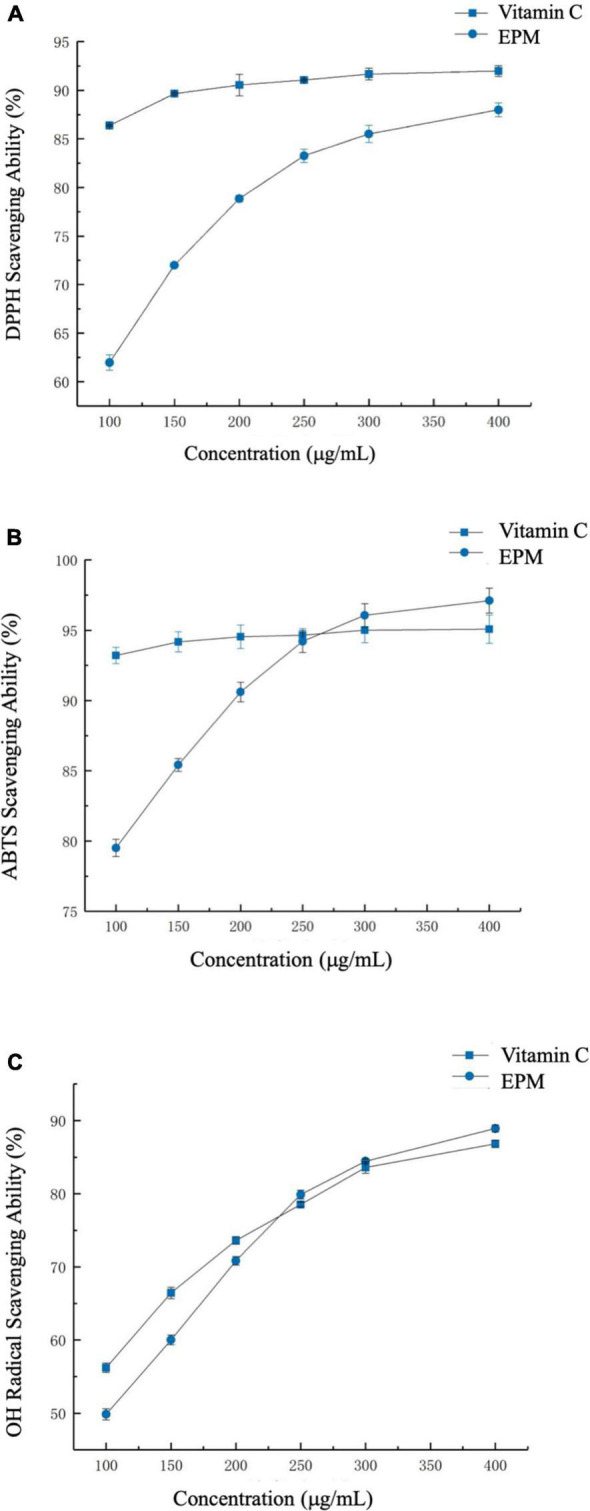
The antioxidant activities of the *Pueraria lobata* extract. **(A)** 2,2-Diphenyl-1-picrylhydrazyl (DPPH) scavenging activity, **(B)** 2,2′-azinobis-(3-ethylbenzthiazoline-6-sulphonate) (ABTS) scavenging activity, and **(C)** hydroxyl radical scavenging activity. CTL, normal control group; EPM, *P. lobata* extract group.

The scavenging activity of DPPH free radical is widely used to evaluate the free radical scavenging capability of natural bioactive compounds (20). All the *P. lobata* extract fractions tested exhibited strong antioxidant activities in this assay in a dose-dependent manner (Figure 2A). For instance, the DPPH free radical scavenging ratio of the *P. lobata* extract was 62.3, 78.8, 85.2, and 88.0% at the concentrations of 100, 200, 300, and 400 μg/ml, respectively. Additionally, the IC_50_ values of DPPH radical scavenging activity by the *P. lobata* extract fraction were 50.8 μg/ml. As well, there were differences in the scavenging ratios exhibited by the different concentrations of the *P. lobata* extract fractions. The antioxidant activity of the *P. lobata* extract was still considerably lower than that of vitamin C at the same concentration. Cherdshewasart et al. found that the same concentration of puerarin (IC_50_ value 93.26 μg/ml) exhibited insignificant antioxidant activity evaluated by DPPH activity as compared with vitamin C (IC_50_ value 72.33 μg/ml) (24). The mechanism of the *P. lobata* extract scavenging DPPH radical could be through providing protons to reduce oxidative free radicals, besides preventing chain reactions of free radicals (25). These findings suggested that the *P. lobata* extract owned a robust antioxidative capacity of scavenging free radicals.

#### Effect of the *P. lobata* Extract on 2,2′-Azinobis-(3-Ethylbenzthiazoline-6-Sulphonate) Radical Scavenging Activity

ABTS free radicals can be inhibited by diverse antioxidant compounds sourced from foods, resulting in the reduced formation of nitrogen-centered ABTS^⋅⁣ +^ cation (26). The *P. lobata* extract scavenged ABTS free radicals in a dose-dependent manner (Figure 2B). For the *P. lobata* extract fraction, the scavenging capabilities were 79.6, 90.7, 96.0, and 97.2% at the concentrations of 100, 200, 300, and 400 μg/ml, respectively. Additionally, the IC_50_ values of ABTS radical scavenging activity by the *P. lobata* extract fraction were 13.9 μg/ml. In addition, the scavenging ability of the *P. lobata* extract was slightly higher than that of ascorbic acid when the concentration of the *P. lobata* extract was over 250 μg/ml. Wang et al. showed that polysaccharides extracted from *P. lobata* exerted a comparative activity to scavenge ABTS^⋅⁣ +^ (27). This capacity of the *P. lobata* extract fraction to scavenge ABTS^⋅⁣ +^ was similar to that result obtained from DPPH radical scavenging assay. All these findings suggested that the *P. lobata* extract, rich in polyphenols, had excellent abilities of scavenging free radicals.

#### Effect of the *P. lobata* Extract on Hydroxyl Radical Scavenging Activity

The antioxidant activities of the *P. lobata* extract were further tested and confirmed by measuring the activity to cleanse hydroxyl radicals. Hydroxyl radicals could attack the biological molecules, interference with normal cellular functions, leading to cause human adverse physical reactions (28). Similar to the results shown above, the *P. lobata* extract (100–400 μg/ml) dose-dependently cleansed hydroxyl radical, which suggested that the *P. lobata* extract could inhibit the production of hydroxyl radical and effectively diminish the oxidative damage in human bodies (Figure 2C). For the *P. lobata* extract fraction, the scavenging activities were 49.7, 70.6, 84.5, and 88.8% at the concentrations of 100, 200, 300, and 400 μg/ml, respectively. In addition, the IC_50_ values of hydroxyl radical scavenging activity by the *P. lobata* extract fraction were 100.4 μg/ml. For comparison, the scavenging capacity of the *P. lobata* extract was higher than that of positive control when the concentration was higher than 225 μg/ml. Consistently, Jin et al. reported that the polyphenols in *P. lobata* riched in daidzein, genistein, B-2-*O*-glucopyranoside, (+)-puerarol, and puerarin could exhibit strong scavenging hydroxyl radical ratios (29). These results denoted that the *P. lobata* extract did excellently in scavenging oxidants through the series of analyses *in vitro*.

### Effect of the *P. lobata* Extract on the Antioxidant Status of Mice Liver *in vivo*

As the mechanisms of antioxidant evaluation methods *in vitro* and *in vivo* were different, in order to determine the potential antioxidative activities comprehensively, mice model was used, and the SOD, GSH-Px activity, T-AOC, and MDA contents in the mice liver were measured, which could be mirrored the cellular antioxidant status as key indicators (Table 1) (30). The main endogenous antioxidant enzymes including SOD, GSH-PX, T-AOC, and MDA in the body system were a protective mechanism, and these compounds could prevent oxidative stress and cell damage (31). Puerarin was reported to possess the potential antioxidative activities both *in vitro* and *in vivo.* The antioxidant effect *in vivo* was partly responded *via* increasing the activities of antioxidant enzymes, for instance, increasing GSH and the ratio of GSH/GSSG in rat liver, as well as reducing the related activity of caspase-3 relative to cell apoptosis (23).

**TABLE 1:** Effects of the *Pueraria lobata* extract on the activities of SOD, GSH-Px, T-AOC, and MDA in the liver of mice, respectively.

	SOD	GSH-Px	T-AOC	MDA
	(U/mg Prot)	(U/mg Prot)	(U/mg Prot)	(mmol/g Prot)
Control	121.2 a	93.1 a	0.81 a	6.2 a
*Pueraria lobata* extract	152.4 b	113.2 b	0.92 b	8.3 b

*The different small letters, respectively, showed significantly different differences between groups (p < 0.05).*

*SOD, superoxide dismutase activities; GSH-Px, glutathione peroxidase peroxide; T-AOC, total antioxidant capacity; MDA, malondialdehyde.*

There was an insignificant difference in the body weight between the control and the *P. lobata* extract groups during the feeding period. The GSH-Px activity in the liver in the *P. lobata* extract group was significantly increased by 21.6% compared with that in the control group (*p* < 0.05). As for the SOD activity, T-AOC and MDA parameters in the mice liver of the *P. lobata* extract group were significantly increased by 25.7, 13.6, and 33.9%, respectively (*p* < 0.05), compared with control. As a result, our study noted that the *P. lobata* extract could enhance the activities of antioxidant enzymes in the liver and inhibit the oxidative damage inside the body, which was evidenced for potentially affecting positively the protection of the liver. Similar findings have been reported by Senevirathne et al. (32). Polyphenols extracted from foods or medical herbs have been also demonstrated a strong ability to scavenge free radicals and terminate oxidative reactions, thereby in consideration of their antioxidant capacities (33, 34). Previously, dietary polyphenol-rich supplementation of barley bran and cranberry (*Vaccinium macrocarpon*) peel could help to boost the antioxidant status of serum and liver based on the mice model evaluation (35, 36). Notably, the results indicated that the enhanced antioxidant capacities of polyphenol-rich extract of *P. lobata in vitro* could be further verified in this mice liver model *via* increasing the activities of SOD, GSH-Px, and MDA, as well as did better in T-AOC than those in the control.

### Effect of the *P. lobata* Extract on the Gut Microbiota

**FIGURE 3:**
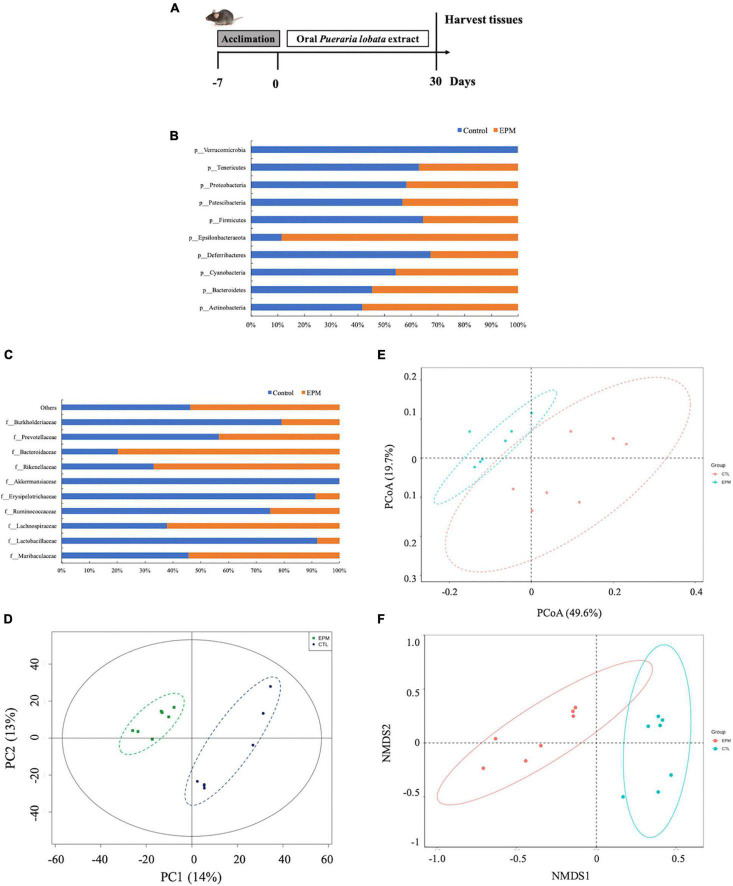
**(A)** Animal design and the antioxidant of the *Pueraria lobata* extract *in vivo*. **(B)** Gut bacterial taxonomic profiling of the control and the *P. lobata* extract groups at the phylum level. **(C)** Gut bacterial taxonomic profiling of the control and the *P. lobata* extract groups at the class level. **(D)** PCA, **(E)** PCoA, and **(F)** NMDS plots of weighted UniFrac and Unweighted UniFrac distance analyses of beta-diversity among samples showing a higher similarity among the bacterial community between the control group and the *P. lobata* extract group.

The *P. lobata* extract was proved to possess the antioxidant bioactivities using *in vitro* and *in vivo* methodologies, and to further investigate the potential effect on the gut microbes, the *in vivo* evaluation of the *P. lobata* extract on the composition and structure of the gut microbiota was performed (Figure 3A). Gut microbiota is responsible for the production of human functional compounds, vitamins, and metabolization of the dietary compound and has a potential beneficial protection against the unhealthy pathogens. Numerous studies showed that any imbalanced status about the composition and structure of gut microbiota would potentially cause the development of colonic dysbiosis, which is powerfully associated with several pathologies and human chronic diseases, including intestinal bowel disease, colon cancer, and obesity (37–39). Polyphenols derived from foods and medicine plants have the capability to reduce bacterial metabolizing enzymes, thereby reducing the colonic tumor incidence and improving cell metabolism; as well, the growth of harmful microbiome could be inhibited, and the abundance of the health-benefit microbiome was enhanced, such as *Lactobacillus* and *Bifidobacterium* species (40). The link of the antioxidant effect of polyphenols *in vivo* and hinder the bacterial proliferation, and the growth of enterpathogenic bacteria was also discussed in several studies (41, 42).

We performed the high-throughput sequencing of the 16S rDNA region to determine the gut microbial community treated by the *P. lobata* extract. The sequencing depth reads of the control and the *P. lobata* extract groups were in the range of 272,642 base pairs (bp) and 295,776 bp, respectively. The data indicated that most of the species in the gut microbiota were captured, and the output of these data was valid. Considering the bacterial change at the phylum level, the microbiome of the control and the *P. lobata* extract groups mainly consisted of *Actinobacteria, Bacteroidetes, Cyanobacteria, Deferribacteres, Epsilonbacteraeota, Firmicutes, Patescibacteria, Proteobacteria, Tenericutes, and Verrucomicrobia* (Figure 3B). The ratios of *Actinobacteria* and *Bacteroidetes* in the *P. lobata* extract group were 1.39-fold and 1.20-fold higher than those in the control group, respectively. However, the ratios of *Cyanobacteria, Firmicutes*, and *Proteobacteria* decreased by 15.5, 44.7, and 28.1% compared with the control group, respectively. The ratio of *Bacteroidetes* and *Firmicutes* phyla consisted of more than 90% population in the gut microbiota, and they would play an important role in human health (43). The *Bacteroidetes* was considered to have an effect to modulate the immune responses and activate the immune lymphocyte T cells (44). In summary, the supplementation of the *P. lobata* extract contributed to the functional differences of mice gut microbiota.

All the 10 most abundant or representative families were *Muribaculaceae, Lachnospiraceae, Lactobacillaceae, Ruminococcaceae, Akkermansiaceae, Erysipelotrichaceae, Rikenellaceae, Bacteroidaceae, Prevotellaceae*, and *Burkholderiaceae* (Figure 3C). The ratios of *Lactobacillaceae, Rikenellaceae*, and *Bacteroidetes* in the *P. lobata* extract group were 1.64-fold, 2.02-fold, and 3.96-fold higher than those in the control group, respectively. However, the ratio of *Ruminococcaceae, Prevotellaceae*, and *Burkholderiaceae* decreased by 66.8, 23.3, and 73.6% than those in the control group. Interestingly, the *P. lobata* extract almost decreased over 90% of *Erysipelotrichaceae* and *Akkermansiaceae* at the family level, which could be useful when these two families of microbiome were overgrown and overproduced in the gut. These results noted that the *P. lobata* extract would be a useful ingredient to restore the balance of the gut ecosystem.

When searching how the *P. lobata* extract and control differ in terms of the composition and structure of gut microbiota profiles, a distance square matrix was calculated to reflect the dissimilarity between the two groups (weighted UniFrac and unweighted UniFrac distances) (Figure 3D). The QIIME software could calculate both weighted and unweighted UniFrac distance, which are phylogenetic measures of beta diversity. We used unweighted UniFrac distance for the PCoA, and the PCoA could help to get principal coordinates and visualize them from complex, multidimensional data (Figure 3E). The results demonstrated that the bacterial communities of the *P. lobata* extract were significantly different from those of the control group. A long-distance between these groups was observed, pointing out that the *P. lobata* extract modulated the bacterial gut microbiome. This distance difference was also significantly evident in the non-metric multidimensional scaling, which displayed the differences among gut microbiota of the *P. lobata* extract and control groups (Figure 3F).

**FIGURE 4:**
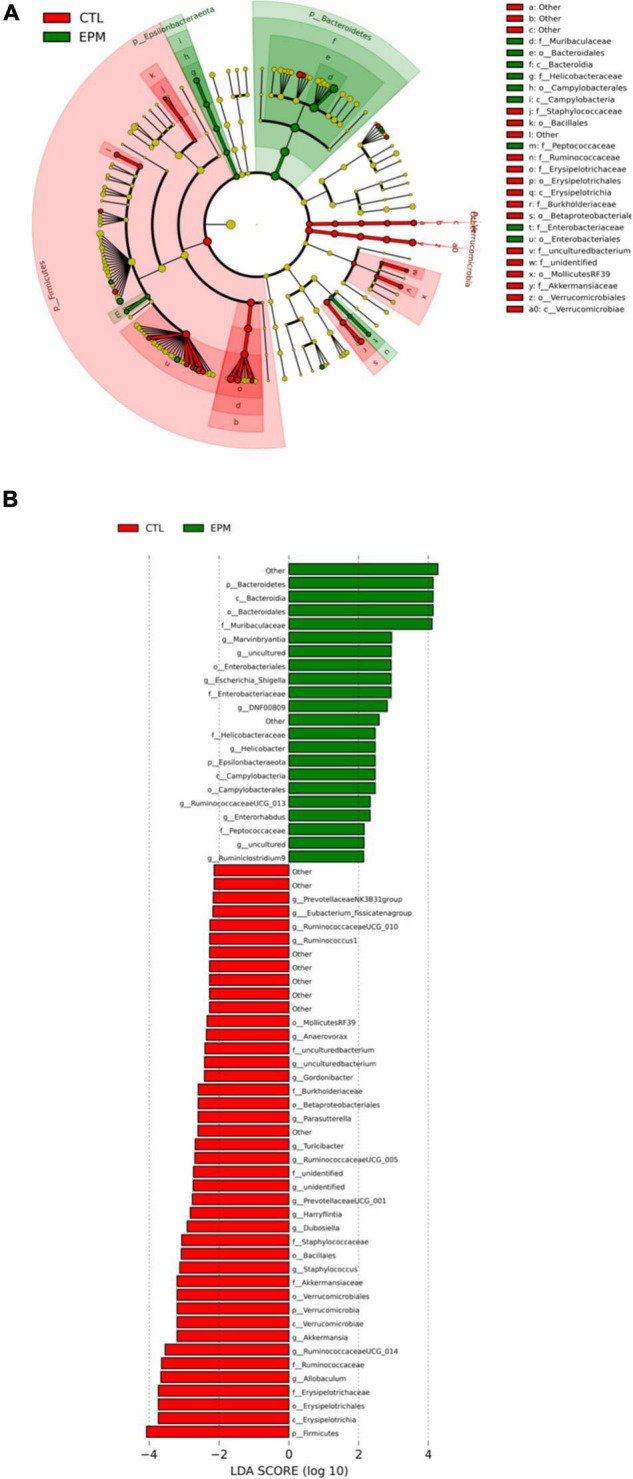
Linear discriminant analysis Effect Size (LEfSe) plot of microbial communities affected by the *Pueraria lobata* extract diet. (Red) CTL; (Green) EPM. The brightness of each dot was proportional to its effect size **(A)**. The taxa had a significant LDA threshold value of > 2 were shown **(B)**. CTL, normal control group; EPM: *P. lobata* extract group.

**FIGURE 5:**
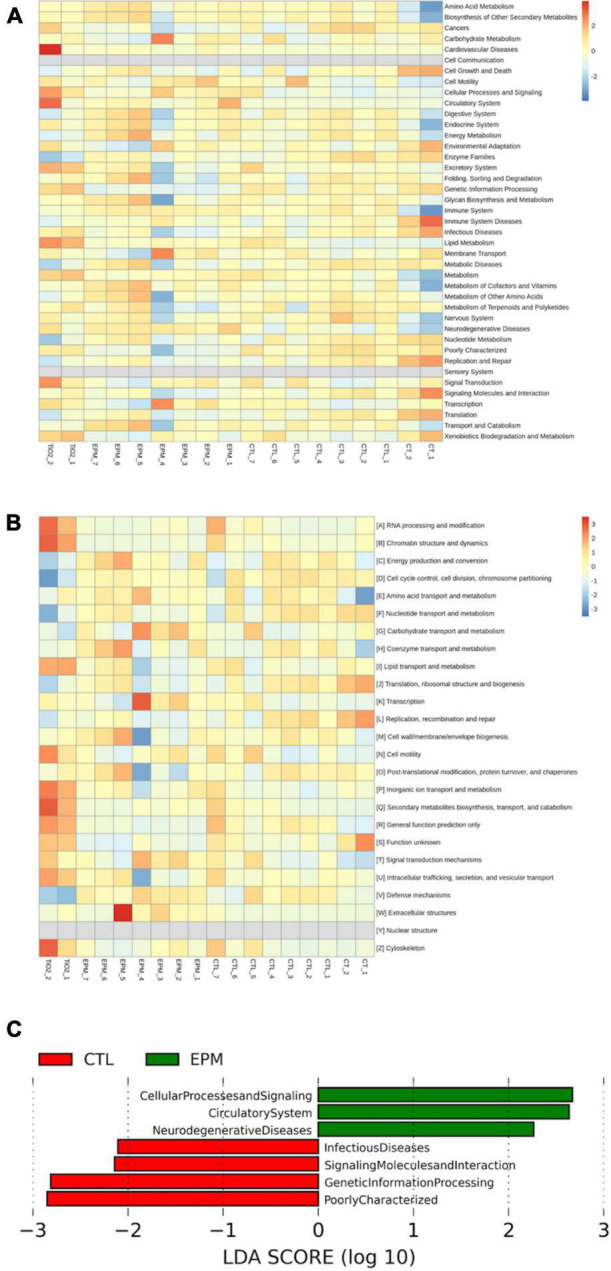
Effect of the normal control group and the *Pueraria lobata* extract on differential operational taxonomic units (OTUs) and predicted microbial community functions by PICRUSt. **(A)** Kyoto Encyclopedia of Genes and Genomes (KEGG) heat map analysis. **(B)** Cluster of orthologous groups of proteins (COG) heat map analysis. **(C)** PICRUSt function prediction. CTL, normal control group; EPM, *P. lobata* extract group.

Altogether, the metagenomics showed the modulation of gut microbiota by the consumption of the *P. lobata* extract group *via* the changing of the abundance of *Firmicutes and Bacteroidetes* phyla and the decrease of *Ruminococcaceae, Verrucomicrobiae*, and *Prevotellaceae* phyla in mice gut (Figures 4A,B). Chen et al. administered 12.5 mg/kg polysaccharides extracted in *P. lobata* to both normal mice and antibiotic-associated diarrhea mice for 2 weeks (45). They showed that the *P. lobata* extract fractions significantly increased the abundance of beneficial bacteria, involving *Oscillospira* and *Anaerotruncus*, whereas relieving colonic pathological changes and gut microbiota dysbiosis additionally. Thus, *P. lobata* polyphenol extract may be a promising source of the phenolic compound that exerts prebiotic effects *in vivo* for human health. In addition to taxonomic composition, the functional profiles were predicted by PICRUSt, and the functional differences are shown in Figure 5. The LEfSe result could illustrate biological explanations to establish the biological consistency and estimation of predicted biomarkers (46). In the treatment of *P. lobata*, the phenolic-rich extracts induced functional differences of microbial communities consisted of cellular process and signaling, circulatory system, and neurodegenerative diseases. This was in consistent with the previous studies that the Puerarin, the main bioactive compound found in *P. lobata*, was found to be beneficial to the neurodegenerative diseases *in vitro* and *in vivo* (47, 48). A similar study found that the bioactive compound puerarin had an obvious anti-inflammatory effect using mice model, and it repaired intestinal mucosal integrity *via* regulating the abundance and kinds of the disorder gut microbiota, increasing the express of tight binding proteins ZO-1 and occludin, and these bacterial *Ruminococcaceae, Lachnospiraceae, Eubacterium_xylanophilum, and Ruminococcus_1* were the key species related (49). This study provides new insight into the potential application of *P. lobata* to be developed as a functional ingredient, which would have a beneficial effect on gut health. The physiological effects of fermentation of *P. lobata* on the potential colon health will be further investigated.

## Conclusion

In this study, we purified the polyphenol-rich extract from *Pueraria lobata* and investigated systematically its antioxidant activities and its effect on gut microbiota. The *P. lobata* extract consisted of a large amount of puerarin that exhibited strong antioxidative capabilities *in vitro* and *in vivo*. Moreover, it had a positive effect on the structure and composition of gut microbiota. In summary, the phenolic-rich extract of *P. lobata* has considerable potential for utilization as a natural antioxidative food additive, as well as a type of gut microbiota-modulating health-promoting food ingredient. Therefore, we suggested that the *P. lobata* extract could be utilized as a promising supplement for functional foods that contributed to the prevention of various human chronic diseases.

## Data Availability Statement

The datasets presented in this study can be found in online repositories. The names of the repository/repositories and accession number(s) can be found in the article/supplementary material.

## Ethics Statement

The animal study was reviewed and approved by the Institutional Animal Care and Use Committee of China Pharmaceutical University.

## Author Contributions

XX conceived the project idea and obtained the funding. XX and CS assigned the project, performed the research, and wrote the manuscript. YG, SC, WM, and XLX finished all the research. JM, JS, and CS revised the manuscript. XX, SH, LJ, and JM participated in the method and review. All authors approved the final version of the manuscript.

## Conflict of Interest

The authors declare that the research was conducted in the absence of any commercial or financial relationships that could be construed as a potential conflict of interest.

## Publisher’s Note

All claims expressed in this article are solely those of the authors and do not necessarily represent those of their affiliated organizations, or those of the publisher, the editors and the reviewers. Any product that may be evaluated in this article, or claim that may be made by its manufacturer, is not guaranteed or endorsed by the publisher.

## Footnotes

1
https://dataview.ncbi.nlm.nih.gov/object/PRJNA805070
